# Alterations of Metabolites in the Frontal Cortex and Amygdala Are Associated With Cognitive Impairment in Alcohol Dependent Patients With Aggressive Behavior

**DOI:** 10.3389/fpsyt.2020.00694

**Published:** 2020-09-11

**Authors:** Chang Liu, Xuefei Tian, Yang Ling, Jiabin Xu, Xuhui Zhou

**Affiliations:** ^1^ Department of Psychiatrics, Brains Hospital of Hunan Province, Changsha, China; ^2^ Clinical Medical School, Hunan University of Chinese Medicine, Changsha, China; ^3^ Clinical Medical Research Center, Hunan Provincial Mental Behavioral Disorder, Changsha, China; ^4^ Department of Psychiatrics, The Ninth Hospital of Changsha, Changsha, China

**Keywords:** alcohol dependence patients, frontal cortex, amygdala, cognitive impairment,¹H MRS

## Abstract

**Background:**

Alcohol dependence (AD) patients have a high prevalence of aggressive behavior (AB). The frontal cortex and amygdala contains various neurotransmitter systems and plays an important role in AB, which is also associated with cognitive deficits. However, to date, no study has addressed the association of metabolites in the frontal cortex and amygdala with cognitive deficits in Chinese aggressive behavior-alcohol dependent patients(AB-ADs).

**Methods:**

We recruited 80 male AD and 40 male healthy controls (HCs), who completed the Repeatable Battery for the Assessment of Neuropsychological Status (RBANS), the Modified Overt Aggression Scale (MOAS), and the proton magnetic resonance spectroscopy (¹H MRS) scan using 3.0T Siemens. The ¹H MRS data were automatically fitted with a linear combination model for quantification of metabolite levels of n-acetyl-aspartate (NAA), glutamate (Glu), Choline (Cho) and creatine (Cr). Metabolite levels were reported as ratios to Cr.

**Results:**

The AB-ADs group scored significantly lower than the non-aggression-alcohol dependent patients (NA-ADs) on these two RBANS subscales (immediate memory and attention function indices). The AB-ADs group showed a significant reduction in NAA/CR ratio in the left frontal cortex and Cho/Cr ratio in the left amygdala, and elevation in Glu/Cr ratio in the bilateral amygdala, compared with the NA-ADs group. The NAA/Cr ratio in the left frontal cortex was positively associated with immediate memory (r=0.60, P<0.05), and the Glu/Cr ratio in the right amygdala was negatively associated with delayed memory (r=-0.44,P<0.05) in AB-ADs group.

**Conclusions:**

Metabolite alterations in the frontal cortex and amygdala may be involved in the pathophysiology of AB in AD and its associated cognitive impairment, especially immediate memory and delayed memory.

## Introduction

Alcohol dependence patients (AD) have a higher rate of aggressive behavior (AB) than patients with other psychiatric disorders, and the general population (1). The frequency of violent behavior in alcohol dependent patients has been reported to range from 20% to 50% (2). Moreover, the AB of AD patients can lead to economic burdens and social problems (3, 4) such as crime, and it predicts poor clinical outcome in AD patients (5). However, not all AD patients exhibit such aggressive behaviour (6) and it is unknown whether specific pathophysiological mechanisms cause AB in AD patients.

Alcohol-related AB is associated with cognitive deficits, these variables include the following: alcohol myopia (narrowed attention), hostile attribution bias (ambiguous social interactions being perceived as hostile (7), and disinhibition (executive functional abnormality) (8). This evidence has suggested that multi-faceted impairment in the cognition process is an important feature of the neuropsychology of AB in AD patients (9)

Many morphometric and functional neuroimaging studies have shown that aggressive individuals have structure and function abnormalities in regions related to the control of emotions, such as the frontal cortex, amygdala, and nucleus accumbens (10, 11). Studies that have used functional magnetic resonance imaging (MRI) have demonstrated that aggressive individuals showed deficient activation of the medial prefrontal cortex and the increased amygdala reactivity (12, 13). Importantly, several prefrontal cortex (PFC) subregions (anterior cingulate cortex) provide an inhibitory signal to the limbic areas (amygdala) (14). Considering the PFC-amygdala as a circuit, it helps to regulate emotional behavior (14). These pieces of evidence suggest that PFC and the amygdala play an important part in the regulation of AB (15, 16). Many neuroimaging studies have also found that long-term exposure to alcohol leads to serious regional brain changes (17), such as in the frontal cortex (18), amygdala (19) and anterior cingulate cortex (ACC) (20), including gray matter volume (GMV) integrity, regional activity, and cerebral glucose metabolism. These studies suggested that AD patients may share alterations in common brain regions with aggressive individuals, however, there is little known about the specific neurological mechanisms underlying AB and whether these cause AD patients to exhibit aggressive behavior.

Taken together, alcohol-related AB may be associated with specific neuroadaptive changes in the frontal cortex and amygdala by impairing various cognitive functions, including prefrontal control of emotional behavior (9, 21). However, no study has explored the association between activation in brain regions and cognitive functions (with imaging or electrophysiological techniques) in AD patients with AB. ^1^ H magnetic resonance spectroscopy (^1^ H MRS) is an invasive method, which can help to examine the metabolites in the brain regions (22). In this present study, we performed this method to explore the quantification of metabolite levels of n-acetyl-aspartate (NAA), glutamate (Glu), Choline (Cho), and creatine (Cr) in alcohol dependent patients with and without AB. We aimed to investigate whether AD patients with AB have specifically altered metabolites in the frontal cortex and amygdala associated with relevant cognitive functions. It was hypothesized that firstly, AB-AD patients would be associated with impaired cognitive functions and altered metabolite levels in the frontal cortex and amygdala; and secondly, that these impaired cognitive functions would be significantly correlated with altered metabolite levels in AB-AD patients.

## Methods

### Participants

Eighty male AD inpatients and forty HCs were recruited from Hunan brain hospital, ChangSha city, Hunan, China. All subjects met the following inclusion criteria: 1) age 18-60 years, Han Chinese; 2) no contraindications for MRI. Participants were excluded if they (i) had any general medical conditions or neurological disorders, including infectious, hepatic, or endocrine disease; (ii) had a history of severe head injury with skull fracture or loss of consciousness of more than 10min; (iii) had any current or previous psychiatric disorder; (iv) had a family history of psychiatric disorder; (v) had contraindications for MRI. Alcohol dependence patients confirmed DSM-IV diagnosis of alcohol dependence and abstained from alcohol for at least 7 days before scanning. AD inpatients were excluded if they met criteria for other substance dependence (excluding nicotine dependence) at any time.

Written informed consent was given by all subjects. This study was approved by the Ethics Committee of the Brain Hospital, Traditional Chinese Medicine University.

### Clinical Characters and Aggressive Behavior Measures

Each subject filled out a detailed questionnaire that recorded general information, sociodemographic characteristics, medical and psychological conditions, and smoking behavior. In addition, we administered an alcohol use questionnaire to record alcohol history and family history of alcohol from each subject. Additional information was collected from available medical records and collateral data (from family and/or treating clinician). The patient’s degree of AB was assessed by the Chinese translation of the standardized Modified Overt Aggression Scale (MOAS). We divided the AD patients into groups based on their AB history and MOAS scores. AD patients with AB were defined as persons who had shown aggressive behavior on more than two instances within half a year, and rated with a score of≥8 in the MOAS. AD patients without AB were defined as individuals who had no AB history and rated with a score of < 8 in the MOAS.

### Cognitive Tests

Cognitive functioning was assessed by the Repeatable Battery for the Assessment of Neuropsychological Status (RBANS, Form A) (23). The RBANS consists of 12 subtests that are used to calculate 5 age-adjusted index scores and a total score. Test indices are Immediate Memory (comprised of List Learning and Story Memory tasks); Visuospatial/Constructional (comprised of Figure Copy and Line Orientation tasks); Language (comprised of Picture Naming and Semantic Fluency tasks); Attention (comprised of Digit Span and Coding tasks); and Delayed Memory (comprised of List Recall, Story Recall, Figure Recall, and List Recognition tasks). The Chinese version of RBANS has good clinical validity and test-retest reliability (24).

### Magnetic Resonance Data Acquisition

**Figure 1:**
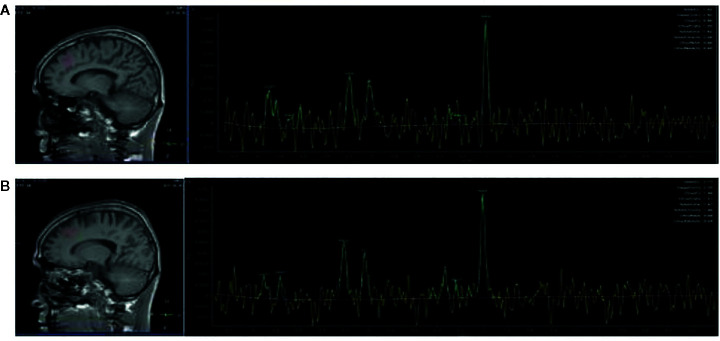
Region of interest in the frontal cortex in views, and spectra of the unfiltered data superimposed with the LCModel fit. **(A)** Region of interest in the left frontal cortex in views, and spectra of the unfiltered data superimposed with the LCModel fit. **(B)** Region of interest in the right frontal cortex in views, and spectra of the unfiltered data superimposed with the LCModel fit.

**Figure 2:**
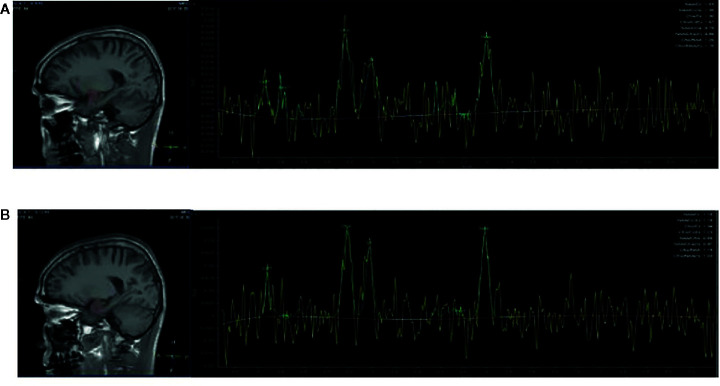
Region of interest in the amygdala in views and spectra of the unfiltered data superimposed with the LCModel fit. **(A)** Region of interest in the left amygdala in views, and spectra of the unfiltered data superimposed with the LCModel fit. **(B)** Region of interest in the right amygdala in views, and spectra of the unfiltered data superimposed with the LCModel fit.

The MRS scan was performed using a Siemens Magnetom Trio Tim 3.0 MR scanner (Siemens, Erlangen, Germany) at the Magnetic Resonance Center of Hunan brain Hospital, China. T1-weighted three-dimensional images were acquired using a gradient echo sequence (repetition time=2,000ms, echo time=2.26ms, field of view=256×256mm, flip angle=8^◦^, matrix size=256×256, number of slices=176, slice thickness=1mm). Using these images, a single^1^ H MRS voxel was placed on the corpus callosum and centered on the intrahemispheric fissure, including frontal cortex (see **Figures 1A, B**), and amygdala (see **Figures 2A, B**).^1^ H MRS was performed using a short-echo point resolved spectroscopy sequence (PRESS;repetition time=1,000ms;echo time=144ms; voxel size 10×10×10mm; number of scans=328). Water suppression was achieved using a chemical shift selective (CHESS) sequence. The area under each peak was measured and the ratio of NAA/Cr to Cho/Cr was calculated using software provided by General Electric Company (GE Funtool2). The position of NAA is determined at the mass fraction of 2.0×10^-6^ on the spectral line, and the chemical shifts of other substances were determined by using it as a reference. The mass fraction of Cho is 3.2×10^-6^ and the mass fraction of Cr is 3.02×10^-6^. In the calculation, the under-peak area of each peak is measured respectively. According to the usual calculation method, taking Cr as the internal standard, the ratio of NAA and Cho to Cr was calculated. All scans were performed by the same radiologist, and all images were recorded on disk for measurement and analysis at the same time.

#### Statistical Analysis

Demographic and clinical variables of the AD inpatients with AB and without AB groups and HCs were compared using t-test, analysis of variance (ANOVA) for continuous variables and chi-squared for categorical variables. We compared RBANS scores and metabolite ratios among the three groups using analysis of variance (ANOVA). Fisher’s least significant difference (LSD) test was used to perform post-hoc pair-wise between-group comparisons. The relationship between the RBANS scores and metabolite ratios was examined by Pearson’s or Spearman’s correlation analysis, followed by the Bonferroni test. In these analyses, all variables were initially entered simultaneously to determine the overall influence, and then backward stepwise procedures were employed to determine the significant associations. SPSS version 19.0 was used to do all statistical analysis. Data were presented as mean and standard deviation (mean ± SD). All p values were 2 tailed with a significance level set at 0.05.

## Results

### Participants Characteristics

The three groups, AB-AD patients (MOAS≥8, n=40), non-aggression-alcohol dependent (NA-AD) patients (MOAS< 8, n=40)and HCs were matched in terms of age, levels of education and the daily number of cigarettes smoked. The AB-AD patients and NA-AD patients were matched in terms of the duration of alcohol use, age of first alcohol use, and the dosage of alcohol used per day. Details of all self-report and behavioral measures are given in **Table 1**.

**Table 1:** Demographic and alcohol use characteristics of alcohol dependent patients with Modified Overt Aggression Scale (MOAS) and health controls.

Characteristic	AB-ADs(n = 40)	NA-ADs(n = 40)	HCs(n = 40)	P
Age (years)	44.35 ± 5.52	41.28 ± 5.23	40.46 ± 6.24	0.10
Education (years)	9.30 ± 2.15	9.45 ± 1.74	9.64 ± 1.94	0.14
Age of first alcohol use (years)	16.55 ± 1.88	16.90 ± 1.93	17.65 ± 1.54	0.11
Alcohol used per day	233.28 ± 24.65	212.56 ± 31.41	–	0.10
Duration of alcohol use (years)	12.25 ± 8.24	10.52 ± 7.51	–	0.20
Number of abstinence	1.23 ± 1.17	0.88 ± 0.95	–	0.08
Cigarette smoked per day	28.50 ± 6.25	30.65 ± 4.55	27.45 ± 6.75	0.12

AB-ADs, aggressive behavior-alcohol dependent patients; HCs, healthy controls; NA-Ads, non-aggression-alcohol dependent patients.

### Group Differences in Cognitive Performance

RBANS index scores of 40 AB-AD patients, 40 NA-AD patients, and 40 HCs are shown in **Table 2**. The AB-AD patient group scored significantly lower than the NA-AD patients on these two RBANS subscales (immediate memory and attention function indices) (p<0.001).

**Table 2:** Index scores on the RBANS in AB-ADs, NA-Ads, and health controls.

Index	AB-ADs(n=40)	NA-ADs(n=40)	HCs(n=40)	p^a^	p^1^	p^2^	p^3^
Immediate memory	30.50 ± 6.37	36.98 ± 6.07	49.43 ± 4.89	0.03	0.02	0.04	0.01
Visuospatial/ constructional	14.98 ± 4.76	16.43 ± 5.14	18.20 ± 4.31	0.12	0.13	0.11	0.12
Language	29.68 ± 5.55	28.68 ± 5.98	32.00 ± 5.51	0.06	0.08	0.07	0.08
Attention	63.03 ± 11.26	70.43 ± 10.62	75.85 ± 11.12	0.13	0.01	0.12	0.02
Delayed memory	55.56 ± 19.30	58.05 ± 12.56	67.23 ± 12.30	0.21	0.04	0.03	0.23

AB-ADs, aggressive behavior-alcohol dependent patients; HCs, healthy controls; NA-ADs, non-aggression-alcohol dependent patients.

p^a^ ANOVA,

p^1^aggressive behavior-alcohol dependent patients vs healthy controls,

p^2^ non-aggression-alcohol dependent patients vs healthy controls,

p^3^ aggressive behavior-alcohol dependent patients vs non-aggression-alcohol dependent patients vs healthy controls.

### ^1^ H MRS Metabolite Ratios

The AB-AD patient group showed a significant reduction in NAA/CR ratio in the left frontal cortex and Cho/Cr ratio in the left amygdala, and elevation in Glu/Cr ratio in the bilateral amygdala, compared with the NA-AD patients group (**Table 3**).

**Table 3:** Metabolite concentrations in the region of interest in AB-ADs, NA-ADs, and health controls.

	AB-ADs(n=40)	NA-ADs(n=40)	HCs(n=40)	p^a^	p^1^	p^2^	p^3^
NAA/CR left frontal cortex	2.27 ± 0.54	3.26 ± 0.78	3.87 ± 0.77	0.038	0.027	0.046	0.04
right frontal cortex	3.18 ± 0.64	3.42 ± 0.56	3.74 ± 0.46	0.12	0.047	0.13	0.11
left amygdala	1.92 ± 0.72	1.96 ± 0.61	2.48 ± 0.74	0.044	0.042	0.043	0.12
right amygdala	1.87 ± 0.55	1.96 ± 0.51	2.03 ± 0.65	0.22	0.18	0.21	0.09
Cho/Cr left frontal cortex	1.24 ± 0.33	1.32 ± 0.45	1.78 ± 0.32	0.045	0.03	0.032	0.10
right frontal cortex	1.15 ± 0.34	1.08 ± 0.56	1.56 ± 0.65	0.03	0.043	0.037	0.08
left amygdala	3.25 ± 1.24	4.56 ± 1.03	4.58 ± 1.14	0.04	0.027	0.035	0.03
right amygdala	3.65 ± 0.55	3.97 ± 0.51	5.82 ± 0.45	0.044	0.038	0.043	0.034
Glu/Cr left frontal cortex	5.21 ± 1.21	5.56 ± 1.32	5.34 ± 1.03	0.14	0.09	0.1	0.11
right frontal cortex	4.85 ± 1.66	4.97 ± 1.56	5.01 ± 1.34	0.11	0.13	0.15	0.14
left amygdala	8.75 ± 3.65	5.06 ± 2.78	4.98 ± 2.65	0.03	0.01	0.07	0.02
right amygdala	7.82 ± 3.25	5.36 ± 2.02	4.54 ± 2.33	0.043	0.03	0.11	0.04

AB-ADs, aggressive behavior-alcohol dependent patients; HCs, healthy controls; NA-Ads, non-aggression-alcohol dependent patients; NAA, n-acetyl-aspartate; Cho, choline; Glu, glutamate; Cr, creatine.

p^a^ ANOVA,

p^1^aggressive behavior-alcohol dependent patients vs healthy controls,

p^2^ non-aggression-alcohol dependent patients vs healthy controls,

p^3^ aggressive behavior-alcohol dependent patients vs non-aggression-alcohol dependent patients vs healthy controls.

### Correlation Between Metabolite Ratios and Cognitive Performance

**Figure 3:**
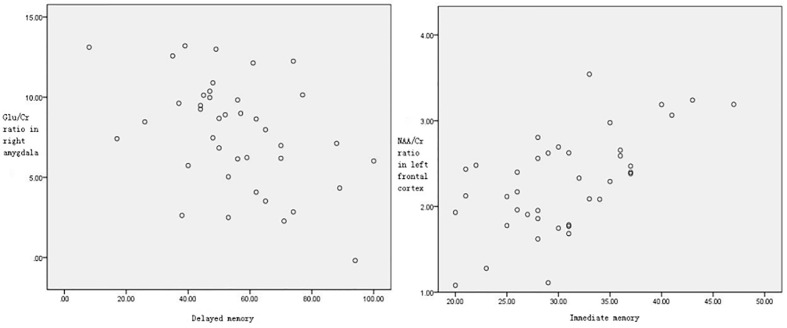
NAA/Cr ratio in the left frontal cortex was significantly correlated with the score of immediate memory index (r=0.60,P<0.05). Glu/Cr ratio in the right amygdala was significantly correlated with the score of delayed memory (r=-0.44, P < 0.05).

For the AB-AD patients, correlation analyses showed a significant positive association between NAA/Cr ratio in the left frontal cortex and the score of immediate memory index (r=0.60,P<0.05); and a significant negative association between Glu/Cr ratio in the right amygdala and the score of delayed memory (r=-0.44,P<0.05) (**Figure 3**).

## Discussion

The main findings of the present study were: (1) significantly lower scores on the two RBANS subscales (immediate memory and attention function indices) were found in AB-AD patients than NA-AD patients; (2) a reduction in NAA/CR ratio in the left frontal cortex and Cho/Cr ratio in the left amygdala, and an elevation in Glu/Cr ratio in the bilateral amygdala of AB-ADs, compared with NA-ADs group; (3) the NAA/Cr ratio in left frontal cortex was positively associated with immediate memory and the Glu/Cr ratio in the right amygdala was negatively associated with delayed memory in AB-AD patients group.

In this study, we found that significantly lower cognitive performance on the two RBANS subscales (immediate memory and attention function indices) were found in AB-AD patients than NA-AD patients. The impairment of attention and memory is associated with an increased manifestation of alcohol-related aggression and has been hypothesized to be an important factor in the onset of alcohol-related aggression (25, 26). Taken together, these results suggest that AB-AD patients had more neuropsychological impairments than NA-AD patients.

Finding the reduced ratio of NAA/CR in the AB-AD patient group is consistent with the theory that substance abuse leads to long-lasting impairment of the frontal cortex and as well as the AD patients (27, 28). Some studies suggested that AD patients have structural and functional abnormalities in the frontal cortex, which is associated with decision-making and inhibition control of AB (9). In addition, NAA reflects neuronal integrity, viability, and number (29). The changes in NAA may reflect adverse neuron function disorder (30). This present finding suggests that there is decreased neuronal integrity, viability, number, or dysfunction in the frontal cortex in AD patients, which may be a neurobiological marker of alcohol-related aggressive behavior.

We also found that the AB-AD patient group showed a significant reduction in the Cho/Cr ratio in the left amygdala. The decreased ratio of Cho/Cr in the left amygdala for the AB-ADs group is in line with previous studies on AD patients (31, 32). Some studies have suggested that Cho is taken as a marker of glial density and represents the status of glial cells in the brain (33, 34). Reduction of Cho/Cr may suggest the proliferation of glial cells due to the damage of neurons induced by alcohol dependence patients (33), which is a marker of AD patients induced neurons damage after Cho decreased. We also found that the AB-AD patient group showed an elevation in Glu/Cr ratio in the bilateral amygdala, compared with the NA-AD patient group, previous studies on alcohol dependent patients have found such findings (35). Glu is the major excitatory neurotransmitter, which is the main precursor and involved in the regulation of brain amino acid metabolism (36). The glutamateric signal system plays an important role in inhibition and behavior control (37). The abnormal function of the glutamate energy system is related to the pathophysiology of mood disorder (38). Thus, our findings, which observed the increased Glu/Cr ratio in the bilateral amygdala, may be associated with an increased manifestation of alcohol-related aggression behavior.

The current findings indicate that metabolite alterations in the frontal cortex and amygdala were associated with worse cognitive function in aggressive behavior-alcohol dependent patients. This finding is consistent with two studies showing that metabolite alterations in the frontal lobes and limbic regions were associated with worse neuropsychological function in AD patients (39, 40). Studies that used as an animal model have discussed that frontal lobe lesion delays memory task completion time (41). Most importantly, frontal lobe lesions have lead to reduced working memory performance in human models (42). These results show that metabolites alterations may play a role in the cognitive process of AB-AD patients, suggesting that alcohol-related aggressive behavior is not just associated with cognitive function impairment but is also related to metabolite alterations in frontal cortex and amygdala.

### Limitations

Several limitations of this study should be noted. First, all subjects in our study were male, making it impossible to explore the influence of gender confidently. Second, our study is cross-sectional, and it is unclear whether alterations in cognition performance and metabolites would persist or be reversed after a continued period of abstinence from alcohol. Finally, this study only focused on the metabolic changes in some brain regions (frontal lobe, amygdala) related to aggressive behavior. In the future, studies using whole-brain analysis may help to provide further insight into the relationship between brain regions and aggressive behavior in alcohol dependent patients. Future studies should also include more women AB-AD patients, and measure alterations longitudinally, examining AB-AD patients for a longer duration, starting from the beginning of abstinence, and covering a longer period of abstinence.

## Conclusion

Our findings suggest that alterations in the ratios of NAA/CR, Cho/Cr, and Glu/Cr in the frontal cortex or amygdala may underline the pathophysiology of neurological impairment in AB-AD patients. These AD patients with AB also displayed a range of cognitive impairments, and metabolite alterations were associated with worse cognitive function in these AD patients. This study indicates that neurotransmitter alteration constitutes a risk factor for cognitive deficits in AB-AD patients. The reduction of NAA/CR in the frontal cortex and Cho/Cr ratio in the amygdala or elevation of the Glu/Cr ratio in the amygdala may be of great clinical significance in treatment.

## Data Availability Statement

The raw data supporting the conclusions of this article will be made available by the authors, without undue reservation.

## Ethics Statement

The studies involving human participants were reviewed and approved by Ethics Committee of the brain Hospital, Traditional Chinese Medicine University. The patients/participants provided their written informed consent to participate in this study.

## Author Contributions

CL, XZ, and XT designed the study. CL, YL, and JX managed the data collection. CL and YL undertook the statistical analysis. CL wrote the first draft of the manuscript. All authors contributed to the article and approved the submitted version.

## Funding

This research was supported by the Diagnosis and Treatment Enhancement Project of Hunan Provincial Severe Mental illness (2018SK70020), and First class Discipline in Clinical Medical School of Hunan University of Chinese Medicine, and a grant of the National Natural Science Youth Found of the Hunan Province (2018JJ3284 to XZ), and a grant of the National Key research and development program (2018YFC1314401 to XZ), and a grant of the Scientific Research Project of Hunan Provincial Health Commission (20200620 to XZ), and a grant of the Hunan Research Center for Mental and Behavioral Disorders (2018SK70020 to XZ), and Provincial technology innovation guide program funded by the Hunan Department of Science and Technology (2017SK50312 to CL), and by Outstanding Young program funded by the Hunan Province Department of Education (18B254 to CL), and a grant of the Ministry of Education of China for Outstanding Young Teachers in University, a grant of the National Natural Science Foundation of Hunan Province (2019JJ80028 to LX).

## Conflict of Interest

The authors declare that the research was conducted in the absence of any commercial or financial relationships that could be construed as a potential conflict of interest.
